# A Case Report of Nongerminal Center B-Cell Type Diffuse Large B-Cell Lymphoma Treated to Complete Response with Rituximab and Ibrutinib

**DOI:** 10.1155/2018/5471368

**Published:** 2018-03-13

**Authors:** Geoffrey Shouse, Miemie Thinn

**Affiliations:** ^1^Division of Hematology and Oncology, Loma Linda University School of Medicine, Loma Linda, CA, USA; ^2^Division of Hematology and Medical Oncology, Loma Linda Veterans Administration Medical Center, Loma Linda, CA, USA

## Abstract

Diffuse large B-cell lymphoma (DLBCL) is a molecularly heterogeneous disease consisting of different subtypes with varying clinical behaviors. For example, the activated B-cell-like (ABC) type of DLBCL has lower cure rates with traditional chemotherapy regimens. The molecular pathway promoting tumorigenic growth of the ABC type includes a dependence on intracellular signaling by Bruton's agammaglobulinemia tyrosine kinase (BTK). This specific pathway has led to the investigation of the utility of ibrutinib in treatment of this type of lymphoma at relapse or in combination with standard chemotherapy. In elderly patients stricken with this disease, standard combination chemotherapy can pose significant toxicity. Some reduced intensity regimens have activity but significantly less favorable long-term outcomes and still pose significant toxicity to elderly patients. In the following case, we demonstrate induction of complete response in an elderly patient with significant comorbidities with nongerminal center B-cell type (NGCB) DLBCL treated with rituximab, ibrutinib, and prednisone. Toxicity included atrial fibrillation that ultimately led to heart failure as well as sepsis which ultimately led to the patient's demise. Despite this fact, the response to treatment appeared durable. This case illustrates the utility and limitations of molecularly targeted therapies to treat aggressive lymphoma in frail elderly patients.

## 1. Introduction

Diffuse large B-cell lymphoma (DLBCL) is a molecularly heterogeneous disease, with multiple subtypes that have variable clinical characteristics. Recent studies indicate that the molecular disruptions in various DLBCL subtypes may explain the observed differences in clinical behavior. For example, the two most common subgroups of DLBCL include the germinal center B-cell-like (GCB) and the non-GCB or activated B-cell-like (ABC) subtypes. The goal of many recent and ongoing studies has been to elucidate the underlying molecular mechanisms promoting cancer growth to identify pathways that can be potentially targeted with less toxic and more efficacious treatments, rather than toxic multiagent chemotherapy. One recent and significant targeted therapy that has revolutionized the treatment of CD20-positive lymphomas is the use of therapeutic anti-CD20 monoclonal antibodies, such as rituximab. The addition of rituximab to standard multiagent chemotherapy has improved survival across all DLBCL subtypes [1].

Despite significant gains in response rates, ABC DLBCL still has a lower rate of cure compared to the GCB type when using conventional R-CHOP chemotherapy [2]. The underlying molecular signaling pathways acting in the ABC subtype of DLBCL are thought to be responsible for this difference. The viability of the ABC subtype of DLBCL is sustained by signaling from the B-cell receptor (BCR) [3]. The BCR is critically important for normal B-cell development and is linked to the development of many of the B-cell malignancies including DLBCL. The BCR is a transmembrane immunoglobulin receptor associated with a heterodimer of CD79a and CD79b. Upon binding of the antigen, the BCR activates the tyrosine kinases LYN and SYK, which initiate a downstream signaling cascade activating intracellular messengers, ultimately leading to increased nuclear factor kappa b (NF-*κ*B) activity, which promotes cell growth and inhibits apoptosis. In the ABC subtype of DLBCL, the NF-*κ*B pathway is constitutively activated by mutations in the BCR and adaptor proteins, as well as the activity of MYD88. The critical link between BCR signaling and NF-*κ*B activation is Bruton's agammaglobulinemia tyrosine kinase (BTK). Signaling from the BCR through LYN and SYK leads to activation of BTK which signals downstream to activate PI3K, phospholipase C2, and the mTOR pathway as well as the mitogen-activated protein kinase ERK, ultimately leading to upregulation and activation of the transcription factor NF-*κ*B [4]. With the advent of the BTK inhibitor, ibrutinib, the potential for targeting this pathway in ABC DLBCL is attractive [5–7]. To date, early phase data indicate the potential for ibrutinib to induce a complete response (CR) in relapsed and refractory DLBCL as a single agent, with the ABC subtype preferentially responding. Studies combining ibrutinib with chemotherapy are ongoing; however, evaluation of a nonchemotherapy combination of both rituximab and ibrutinib has not been described to date [8, 9]. In the present case, we report an elderly patient with significant comorbidities, who was deemed not a candidate for standard therapy and was treated to CR with a combination of rituximab, ibrutinib, and prednisone.

## 2. Case Presentation

The patient was a 70-year-old Caucasian male with coronary artery disease, sick sinus syndrome with pacemaker dependence, chronic kidney disease, type 2 diabetes mellitus, dementia, and schizoaffective disorder, who was found to have spontaneous unilateral epistaxis and left-sided facial asymmetry. He lived at a board and care facility, and medical decisions were made in conjunction with the patient and his sister who was his power of attorney. Prior to these symptoms, the patient was Eastern Cooperative Oncology Group functional status (ECOG) of 2. Upon admission to the hospital, he was found to have a large nasal mass, palpable large left cervical lymphadenopathy. Timeline of events is outlined in Table 1.

**Figure 1:**
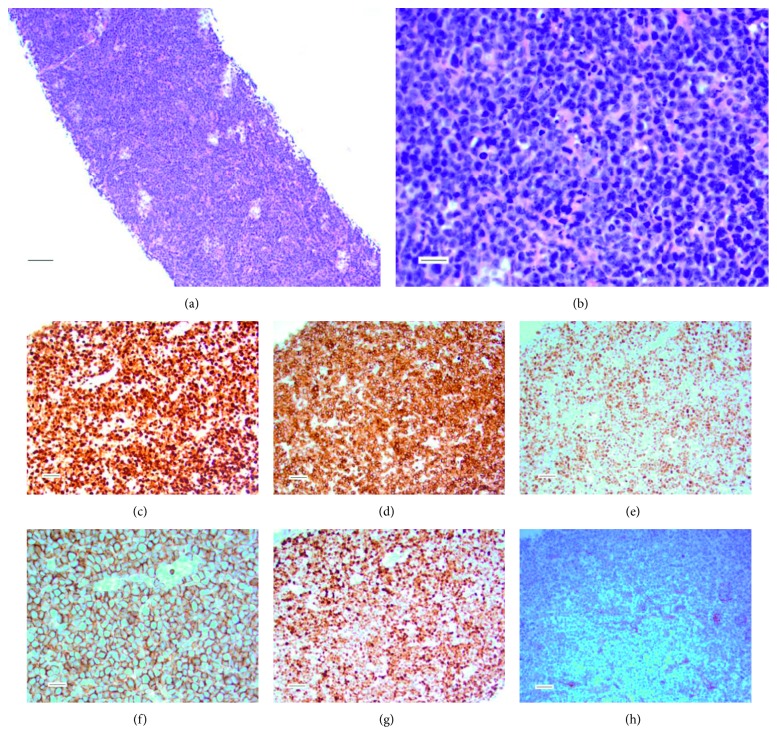
Pathology demonstrating staining consistent with ABC type DLBCL. Hematoxylin and eosin staining demonstrating large lymphocytes with active mitoses at both (a) 100x magnification with bar representing 100 microns and (b) 400x with bar representing 25 microns. (c) Ki-67 stain demonstrating high proliferative index. Various stains including positivity for (d) Bcl2, (e) Bcl6, and (f) CD20 magnified at 400x with bar representing 25 microns and (g) MUM1 as well as negativity for (h) CD10. All images are obtained at 200x magnification with bar representing 50 microns unless otherwise documented.

**Figure 2:**
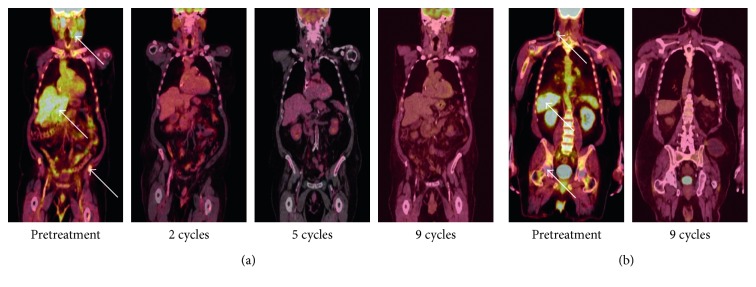
Staging imaging demonstrating response to treatment. (a) PET/CT imaging at initiation of treatment, after 2 cycles of treatment, 5 cycles of treatment, or 9 cycles of treatment as listed. (b) PET/CT imaging showing activity in the bone marrow and liver prior to treatment that was reduced to background levels after 9 cycles of treatment. White arrows indicate areas of active disease on the PET/CT.

CT imaging demonstrated a large infiltrative enhancing mass centered in the left side of the nasal cavity and left maxillary sinus as well as an enlarged left submandibular lymph node. Left submandibular lymph node fine-needle biopsy demonstrated sheets of lymphocytes with effacement of the normal lymph node architecture, with medium to large lymphocytes with nucleoli and vesicular chromatin. By immunohistochemistry, the atypical cells were Pax-5, CD20, MUM1, BCL2, and BCL6 positive and CD56, TdT, CD10, and cyclin D1 negative, with a Ki-67 of about 95%, consistent with diffuse large B-cell lymphoma, NGCB type (Figure 1). PET/CT was performed after the biopsy to complete staging, which demonstrated multifocal hypermetabolic tumor foci including bilateral nasopharynx, bilateral nasal cavity, left maxillary sinus with extension to the left parapharyngeal space, soft palate, lingual tonsil, palatine tonsil, left face, bilateral neck nodes including jugulodigastric, posterior cervical, and the largest of which was a left submandibular node with central necrosis of 4 centimeters (Figure 2(a)). In addition, the liver and bone were diffusely increased in avidity suggestive of involvement by lymphoma (Figure 2(b)). The initial standardized uptake value (SUV) of the left submandibular node was 6.4, the liver was 3.2 SUV, and the bone marrow was 3.7 SUV. The patient and his sister refused biopsy of these sites to confirm the presence of disease pathologically. Based on these findings and a normal initial LDH level of 159 units per liter, he was considered stage IV, with International Prognostic Index (IPI) of 4 (high risk), as well as high risk for CNS involvement. After discussion with the patient and his sister, decision was made to pursue only “nonchemotherapy” treatments. As such, monoclonal antibodies, targeted therapies, and corticosteroids were all considered; however, intrathecal chemotherapy was declined.

The patient started treatment with ibrutinib, and the dose was escalated over several weeks to 420 mg daily by mouth. At the same time, 21-day cycles of rituximab 375 mg/m^2^ IV on day 1 along with prednisone 1 mg/kg PO for days 1 through 5 were also initiated. Supportive care was initiated simultaneously with allopurinol for tumor lysis prophylaxis. In addition, since the patient was wheelchair bound and at risk for thromboembolism, he was started on prophylactic dose of enoxaparin at 40 mg subcutaneously daily. Baseline hepatitis B, C, and HIV testing was negative. The patient tolerated the treatment well with no hematologic toxicities, minimal tumor lysis, no effect on renal or liver function, and no significant infection. The patient had an excellent response to treatment, and after the first cycle of chemotherapy, his epistaxis had resolved and his pain and swelling had also subsided. After five cycles of treatment, the patient had repeat PET/CT imaging that demonstrated near-complete response (Figure 2(a)). The dominant submandibular lymph node decreased in size from more than 4 cm to less than 1.5 cm, there were only residual erosive changes in the maxilla with mild residual FDG avidity (SUV max 3.6), the FDG activity in the liver resolved to SUV 2.7, and however the bone marrow remained PET avid with SUV 3.8. After 4 additional cycles, the patient achieved a complete response on repeat PET imaging including resolution of the bone marrow avidity (Figure 2(a)). This response was durable, and even after cessation of therapy, he had no signs of recurrence.

After a total of 9 cycles of rituximab, prednisone, and ibrutinib, the patient had a complete response (Figure 2). At that time, however, ECG demonstrated that he was in atrial fibrillation with rapid ventricular response. He was referred to cardiology, and his dose of enoxaparin was increased to a therapeutic level at 1 mg/kg subcutaneously twice per day. He was started on beta blockade with control of his heart rate. At that point, the plan was to transition to rituximab maintenance every 2 months. Ibrutinib was continued as his atrial fibrillation seemed to be rate controlled. Repeat PET/CT showed the disease was still in remission one month later; however, prior to his first dose of maintenance rituximab, he presented to an outside hospital with fever, cough, shortness of breath, and atrial fibrillation with rapid ventricular response. He was diagnosed simultaneously with sepsis from pneumonia and respiratory failure from both pneumonia and an acute exacerbation of heart failure. During the hospital course, the patient and his sister decided to stop all interventions and he was made comfort care. He died a few weeks later on hospice care. Despite being off of treatment for several weeks, however, there was no sign of recurrent disease at that time.

## 3. Discussion

In this study, we report a case of stage IV NGCB DLBCL in a patient with significant comorbidities who was treated to CR using 9 cycles of ibrutinib, rituximab, and prednisone. The patient had an excellent clinical response to treatment with resolution of epistaxis and decrease in pain and swelling of his face after the first cycle. His response was durable lasting almost a full year with no evidence of recurrence noted even when he was off therapy for several weeks. The patient initially tolerated the treatment very well with remarkable resolution of tumor burden and minimal effects on blood counts, renal function, liver function, and without significant bleeding, thrombosis, or infection. Of note, however, after achieving complete response, the patient developed atrial fibrillation, a known complication associated with ibrutinib therapy. In addition, at the same time he was noted to have acute onset of systolic heart failure with a drop in ejection fraction from 60% measured prior to treatment to 35%. Although the patient appeared to have the atrial fibrillation under control, after a nonneutropenia-associated infection led to sepsis, it was too overwhelming for his body to overcome. It was likely that ibrutinib was associated with both the atrial fibrillation and the sepsis, as ibrutinib blocks BTK signaling in normal B-cells which may predispose to infection-related complications. Ibrutinib had been stopped for several weeks due to the presumed cardiac toxicity, but despite this, the patient was still without evidence of lymphoma at the time of his death. No other significant dose-limiting toxicities were encountered during his treatment. Review of the literature demonstrates several case reports outlining chemotherapy regimens that have been effective at treating elderly patients, even though these lower toxicity regimens carry with them significant risk and toxic side effects [10–12]. In each of these reports, there were significant toxicities associated with treatment in order to achieve clinical response. Rituximab has been shown to add survival benefit to many CD20-positive B-cell malignancies [13]. Side effects and toxicities were generally mild and included infusion reactions and hypogammaglobulinemia in long-term use. Ibrutinib has demonstrated excellent responses in various hematologic malignancies including chronic lymphocytic leukemia and mantle cell lymphoma [14]. In general, ibrutinib was well tolerated with bleeding and atrial fibrillation being the most common significant side effects. Despite these, in general the medication was well tolerated even in elderly patients over the age of 70. Our case is unique in that our patient was not only elderly but had borderline poor performance status and significant comorbidities. Our patient also had very high-risk disease, stage IV, with indication of both liver and bone marrow involvement based on PET/CT imaging. Finally, it was also unique in that classical cytotoxic chemotherapy was not administered at all. Of additional note, although the patient had significant risk for development of CNS disease, there was no clinical evidence of this occurring on exam or imaging. It is not clear whether this is due to effectiveness of ibrutinib at controlling CNS disease or some other unknown factors, although it was a very encouraging outcome, regardless.

In the field of cancer research and therapeutics, there is a steady movement toward the study, implementation, and use of targeted therapies to treat cancer. These therapies can be tailored to the particular molecular aberrations inherent to a specific subtype of cancer. The goal of these targeted therapies is to improve clinical outcomes, decrease toxicity, enhance response rates, and decrease relapse. In the case of DLBCL, recent studies indicate that the ABC subtype of lymphoma has an addiction to the driving signal from the BCR to NF-*κ*B mediated by BTK. This yields this particular subtype of DLBCL to targeted therapy with ibrutinib. Although initial early phase studies have demonstrated good responses in relapsed and refractory DLBCL, there are limited data to indicate the efficacy of low-toxicity regimens such as that described in this case using rituximab, prednisone, and ibrutinib. The clinical response seen in this patient suggests that in elderly patients with significant comorbidities, complete responses can be achieved, leading to improvement in quality of life and likely duration of life as well. It is important to remember, however, that with new therapies come new important side effects and toxicities. In our case, the patient developed atrial fibrillation, a known toxicity associated with ibrutinib. He also developed uncompensated heart failure related to his atrial fibrillation as well as sepsis which was most likely related to ibrutinib use. It is therefore important to keep important toxicities of these medications in mind, especially in elderly patients with comorbidities. Additionally, it is important to be aware of the potential shortcomings of novel therapies. For ibrutinib in ABC DLBCL, there are known resistance mechanisms. It is important to be aware of these as they may potentially be associated with recurrent disease or disease that is primary refractory of ibrutinib therapy. One such pathway includes the PI3K-Akt-mTOR signaling cascade, either by overexpression of CD79B BCR subunit or mutations in the PLC gamma subunit [15, 16]. In vitro studies suggest these resistance mechanisms can be overcome by inhibition of the PI3K pathway and may represent a second potential target in ABC DLBCL [4]. It will be interesting to see the changes to treatment paradigms in the coming years as these new targeted agents are utilized to their full potential.

## Figures and Tables

**Table 1:** Timeline of major events.

Date	Event
2/13/16	Patient admitted with large nasal mass found
2/13/16	CT demonstrated large infiltrative mass
2/13/16	Left mandibular lymph node fine needle biopsy demonstrating DLBCL NGCB type
3/10/16	Initial staging PET/CT
3/11/16	Cycle 1, day 1 of ibrutinib and rituximab
8/15/16	PET CT showing CR
10/6/16	Cycle 9 of ibrutinib and rituximab completed
10/6/16	First documentation of atrial fibrillation noted
10/17/16	Patient presented with heart failure and sepsis
10/24/16	Patient went to hospice
